# Internet Gaming Disorder: Evidence for a Risk and Resilience Approach

**DOI:** 10.3390/ijerph19095587

**Published:** 2022-05-04

**Authors:** Wayne A. Warburton, Sophie Parkes, Naomi Sweller

**Affiliations:** School of Psychological Sciences, Macquarie University, Sydney 2109, Australia; sparkes2805@gmail.com (S.P.); naomi.sweller@mq.edu.au (N.S.)

**Keywords:** Internet Gaming Disorder, Problematic Video Game Use, Hazardous Gaming, unmet needs, risk and resilience

## Abstract

Although previous research has noted a range of factors that predict developing Problematic Video Game Use (PVGU) and Internet Gaming Disorder (IGD), few studies have looked at risk and protective factors together, and there is scant empirical evidence examining whether risk for PVGU or IGD increases or decreases as risk or protective factors accumulate in the individual. The aim of the current study was to examine both issues using predictors from three demonstrated PVGU and IGD risk categories: executive dysfunction, unmet needs in everyday life, and unhelpful family environment. In a survey of N = 866 12–17-year-old school students, the risk/protective factors that most strongly predicted severity of IGD symptomology and meeting IGD diagnostic criteria were self-control and social exclusion. Other significant predictors included impulsivity, self-esteem, mastery, control of one’s external environment, and better parent-child attachment quality. Trend analyses revealed a linear increase in the risk of PVGU as risk and net-risk factors accumulated, and a decrease as protective and net-protective factors accumulated. Thus, a net accumulation of issues around impulse control and unmet needs in everyday life may predispose adolescents to PVGU or IGD. Results support a ‘risk and resilience’ approach to adolescent screen-based disorders and suggest potential benefits to a risk factor focus in treatment.

## 1. Introduction

Although playing video games is a harmless and enjoyable pastime for the vast majority of users, a small minority of players seem to develop serious problems related to their gaming [1]. Formal diagnoses for gaming-related disorders are relatively recent, with Internet Gaming Disorder (IGD) noted as a disorder requiring further research in the 2013 5th edition of the Diagnostic and Statistical Manual for Mental Disorders [2], and Gaming Disorder (GD) and Hazardous Gaming (HG) listed in the 2019 11th edition of the International Classification of Diseases [3].

These listings have been controversial. For example, some scholars have argued that the research base is low in quality, that there is a lack of standardisation in measurement, that the presence of common co-morbid disorders may indicate disordered gaming is secondary to other disorders rather than a disorder in its own right, and that creating such a disorder may stigmatise the many gamers who play without any negative consequences [4]. Others have argued that “most complex mental illnesses have been through a similar process of clarification, research, and movement before there was agreement about the exact diagnostic criteria” but that “they were still classified as a disorder” [5] (p. 3), that ‘pure’ disorders without co-morbidities are the exception rather than the rule in mental health [5] (p. 2), and that having a diagnosis available may actually help to avoid pathologising gamers, by clearly separating those with problems from those without, in much the way that depression is distinguished from passing sadness or that those with eating disorders are separated from those who may diet or over-exercise sometimes [5]. Although it is clear that further evidence and standardised measures are clearly needed, and there is still controversy around IGD and GD diagnoses, there also appears to be substantive and growing clinical and neuroscience evidence that some individuals do develop mental health problems around their video game (VG) use, and that, similar to gambling behaviours, these can occur on a continuum from ‘at-risk’ to ‘hazardous/problematic’ to having multiple sequelae of true addiction [6]. For reviews see [1,7,8,9,10,11,12].

Establishing the prevalence of problematic or disordered video game use has been hampered by the use of differing criteria and measures, but cross-culturally it seems that problems that are clinically subthreshold but substantial—characterised as Hazardous Gaming by the WHO and more commonly referred to as ‘problematic’ video game use (PVGU)—likely have a prevalence of around 5–10% in adolescents and young adults [13,14]. Estimates of adolescents/young adults meeting clinical thresholds are typically 1–3% for IGD [9] and ~2% for GD when stringent sampling criteria are applied [15]. Adolescents, the focus of this study, have the highest prevalence rate of any age group [9] and are perhaps the demographic most psychologically vulnerable to screen-based addictions [16]. A recent study that followed mid-teens into young adulthood found that ~10% had persistent/growing levels of PVGU across six years, with ~2% demonstrating clinical levels of IGD both at baseline and after six years [17]. Given these reasonably high prevalence rates, and the substantial impairments linked with gaming-based disorders [10], it seems important to establish likely risk and protective factors.

In doing this, it is important to be able to establish whether an individual’s video gaming reaches a threshold for being disordered, or if they are at the more severe end of the symptom spectrum, indicating use that is sub-clinical but with the potential to become disordered. Whilst GD is a recognised disorder, it has only recently become so, and screens and measures for GD are still undergoing the process of being validated across multiple populations. In contrast, there are a number of well-validated scales that measure IGD. Thus, the current study focuses on risk and protective factors related to IGD and to higher levels of IGD symptoms, rather than to GD. Although a number of studies have examined IGD risk factors, few (if any) have examined risk and protective factors in the same study, and then determined the cumulative net effect of those factors.

### 1.1. Risk and Protective Factors for IGD

Males are typically more likely to develop IGD than females [18], and a recently published study found a male to female ratio of 2.5:1 for GD [15]. However, age effects are less clear. Research tends to find a peak in IGD during the adolescent years [9], but not always [19]. In terms of non-demographic factors, the IGD literature suggests at least four substantive categories of risk: executive dysfunction/self-control deficits, key needs being unmet in everyday life, an unhelpful family environment, and pre-existing mental health conditions [10,11]. This study examines the first three, which relate to IGD risk in the general population, but does not address the latter, which requires substantial corroborating diagnostic evidence.

Previous research has found strong links between screen-based addictions and dysexecutive problems [9], low self-control [20], poor self-regulation [21], and impulsivity [21,22,23,24]. A similar pattern of risk is also found around unmet needs. Several IGD models focus on the three needs posited by self-determination theory (competence, autonomy and relatedness) and suggest that when these needs are unmet offline but satisfied by video games, the urge to play those games can become problematic [25,26]. Substantial evidence supports the importance of these (and other) needs. Those with IGD, or with a symptomology suggestive of problematic levels of VG use, tend to have fewer friends [27], be lonelier [16,24], have impaired social relationships [28], experience social problems [19], and be more likely to have social phobia/anxiety [24,29]. In terms of competence and mastery, those with IGD tend to have limited non-gaming leisure or recreational activities [19,30,31], and lower educational or academic achievement [19]. In terms of autonomy (and the related need for control of one’s environment), the need to experience power and autonomy has also been implicated in IGD [9]. In addition, low self-esteem seems to be present in many with IGD or with PVGU [22,24], although a recent longitudinal study found that self-esteem may have been more a consequence than a predictor of IGD [32], suggesting further clarification for low self-esteem as a risk factor is needed.

With regards to family environment (which may also impact need satisfaction and self-regulation capability), a number of findings underline its importance in regards to IGD and PVGU. Low levels of parental competence, poor supervision, and inconsistent parenting may all play a role in the development of IGD [9,33,34]. In addition, Kwon and colleagues found that parental hostility was positively related to PVGU and parental affection was a protective factor [33]. Two other studies have found similar effects, but only at a bivariate level. Mößle and Rehbein found that PVGU correlated with lower levels of parental care and higher levels of family violence [35], and King and Delfabbro found that a poor parent–child relationship predicted being in a group at higher risk for IGD, in bivariate but not multivariate analyses [7].

### 1.2. Risk and Resilience in IGD

‘Risk and resilience’ approaches examine whether the risk for an outcome increases as the number of risk factors increase, or decreases as protective factors increase, and have been used successfully in other domains of research such as aggressive behaviour (e.g., [36,37,38,39]). Such an approach seems appropriate for IGD [13] and may have significant clinical implications. For example, if IGD risk increases in a linear or curvilinear fashion as the number of risk factors increase, then reversing the influence of one or more risk factors (for example bolstering executive function) may significantly reduce the risk for developing or maintaining IGD. Thus, as Coyne and colleagues note, understanding such risk and protective factors may be important with regard to the development of intervention programs [13].

### 1.3. The Current Study

The current study was conducted in a population of 12–17-year-old adolescent school students and had the following aims: to determine the risk and protective factors associated with (a) meeting criteria for an IGD diagnosis and (b) higher levels of IGD symptom severity; to establish the strongest predictors; and to use linear modelling to determine if risk increased or decreased in a systematic way as risk or protective factors accumulated in the individual. The following hypotheses were made:

**Hypothesis** **1** **(H1).** *That all measured risk and protective factors would significantly predict both IGD severity scores and meeting diagnostic criteria for IGD at the univariate level*.

**Hypothesis** **2** **(H2).** *That the strongest predictors in multiple regression models would include indicators of key needs not being met and deficits in executive function such as impulsivity and/or low self-control*.

**Hypothesis** **3** **(H3).** *That an accumulation of risk factors would increase the risk of IGD severity scores and the risk of meeting criteria for IGD diagnosis, with the former being demonstrated by a significant linear trend using trend analyses and the latter by increased odds of meeting IGD criteria with each additional risk, using logistic regression.*

**Hypothesis** **4** **(H4).** *That, using similar analyses to Hypothesis 3, an accumulation of protective factors would decrease the risk of IGD severity scores and the risk of meeting criteria for IGD diagnosis.*

**Hypothesis** **5** **(H5).** *That, using similar analyses to Hypotheses 3 and 4, an accumulation of net risk factors (i.e., the residual number of risk factors once protective factors are subtracted) would increase the risk of IGD severity scores and the risk of meeting criteria for IGD diagnosis.*

## 2. Materials and Methods

### 2.1. Participants

Participants were 979 12–17-year-old school students at a large Australian public school with above-average social advantage indicators (i.e., a higher than average Index of Community Socio-Educational Advantage score of 1127, using the Australian Curriculum, Assessment and Reporting Authority system). Data from 113 respondents who completed the survey in an improbably short time (<5 min) or produced data that appeared random or patterned were removed, leaving a final sample for analysis (N = 866) with a mean age of 14.12 (SD = 1.22) that was 57% male and 43% female. In total, 27% of students were in Year 7, with 24%, 28%, and 21% in years 8–10, respectively. The largest ethnic origin groups were those who identified as White/Caucasian (33%), Asian (25%), and from India and the subcontinent (25%).

In terms of the 24 participants who met criteria for IGD, 15 identified as male and 9 as female. This subgroup was of mixed ethnicity—eight identified as White/Caucasian; four as from India and the subcontinent; three as Asian; two as Arabic/Middle Eastern; and one each as Aboriginal/Torres Strait/Pacific Islander and Hispanic/South American. Their ages ranged from 12 to 16, with a mean age of 14.42 (SD = 1.25). In terms of key characteristics, Table 1 shows the means for both this sub-group that met Criteria for IGD and for the overall sample, as well as providing *t*-tests to establish where differences are significant.

### 2.2. Measures

#### 2.2.1. Key Outcomes: Internet Gaming Disorder (IGD) Symptom Severity; Meets IGD Diagnostic Criteria

The 10-item Internet Gaming Disorder Test (IGDT-10) [40] uses a single item to test eight of the nine DSM-5 criteria for IGD [41] (e.g., “*Have you tried to keep your family, friends or other important people from knowing how much you were gaming or have you lied to them regarding your gaming?*”), and two items to measure the more complex final criterion related to impacts on relationships and opportunities. A 3-point Likert scale is used (0 = never, 1 = sometimes, 2 = often), with a score from 0–20 indicating the severity of IGD symptomology. In analyses this is denoted as “IGD symptom severity”.

To ascertain a diagnosis of IGD, ‘sometimes’ responses are recoded to zero and ‘often’ responses to one, with questions 9 and 10 having a maximum total score of one between them. A score of five or more out of nine indicates meeting diagnostic criteria for IGD. In analyses, this is denoted as “meets IGD criteria”.

The IGDT-10 has satisfactory internal consistency, demonstrated construct validity [40] and was noted by King and colleagues (2020) as being one of five IGD measures with greater evidentiary support [42].

#### 2.2.2. Predictors

It is important to note that dimensional predictors may confer risk at one end of the continuum and protection at the other (that is, high scores on a risk factor such as impulsivity may confer risk whilst low scores confer protection; in contrast, for factors such as self-control, higher scores may confer protection and low scores may confer risk). Thus, for clarity, dimensional predictors will be categorised as risk factors where higher levels of that factor confer greater risk, and protective factors where higher levels confer greater protection.

##### Parental Boundaries on Video Game Use (Protective Factor)

Three items asked students who played video games whether parents placed boundaries on their use in relation to time limits, duration limits, and content limits, using a *Yes/No* format.

##### Self-Esteem (Protective Factor)

The widely-used 10-item Rosenberg Self-Esteem Scale (RSES) [43] assesses global self-esteem on a 4-point scale where 1 = *strongly agree* and 4 = *strongly disagree* (e.g., “*I take a positive attitude toward myself*”). Half of the items have a positive valence, and half a negative valence, so in order for higher scores to indicate greater self-esteem, items 1, 3, 4, 7 and 10 were reverse-scored. In line with Rosenberg [43], scores below 15 were taken to indicate low self-esteem. The RSES has demonstrated internal consistency and reproducibility [43].

##### Parent–Child Attachment Quality (Protective Factor)

The 12-item parental attachment subscale of the Modified Inventory of Parent and Peer Attachment (MIPPA) [44] uses a 4-point scale (1 = *almost never or never*; 4 = *almost always or always*) to assess communication, trust, and alienation in parental relations (e.g., “*I tell my parent/s about my problems and troubles*” for communication). Higher scores denote better parent–child attachment quality. The MIPPA has demonstrated internal consistency [44] and predictive validity.

##### Family Connectedness and Family Warmth (Protective Factor)

The 6-item Parent-Family Connectedness Scale (PFCS) [45] measures perceived closeness in the immediate family and the parent–child relationship. Two additional items were added to ascertain perceived feelings of love and warmth between parent and child: “*How much do you feel loved by your parent/s?*” and “*How much do you feel that you have a warm relationship with your parent/s?*” Responses on a 5-point scale (1 = *Not at all*; 5 = *Very much*) were averaged to give a mean score from 1–5. The item additions seemed justified, with the final 8-item scale having excellent internal consistency (α = 0.92).

##### Social Potency (Protective Factor)

The 15-item Social Potency Subscale of the Multidimensional Personality Questionnaire (MPQ) [46] assesses social dominance, persuasiveness, and leadership (e.g., “*I am quite effective at talking people into things*”). It has a *True/False* format, with higher scores indicating greater social potency. This subscale has good to excellent internal consistency [46].

##### Feeling in Control of One’s External Environment (Protective Factor)

A 10-item scale was created to assess levels of perceived control over one’s own external environment (full scale in Appendix A). Participants endorsed statements such as “*My family include me in decision making*” and “*I often feel helpless when dealing with the problems in my life*” using a 6-point scale (1 = strongly disagree; 6 = strongly agree), with higher scores indicating greater perceived control of one’s external environment. Internal consistency was α = 0.80 in this sample.

##### Self-Control (Protective Factor)

The 13-item Brief Self-Control Scale (BSCS) [47] measures typical self-control behaviours (e.g., “*I have a hard time breaking bad habits*”) using two response anchors (1 = *Not at all like me*; 5 = *Very much like me*). High overall scores indicate greater self-control. The full BSCS has good internal consistency (α = 0.83–0.85) and test-retest reliability (α = 0.87), and demonstrated construct validity [47].

##### Mastery (Protective Factor)

Participants were asked to write a list of things they are ‘really good at’. Qualitative responses were re-coded to reflect the count of discrete types of activities that participants reported mastery of, with multiple responses related to a similar skill (e.g., video games Fortnite, Minecraft, Roblox) being reduced to a single count. Participants were then allocated a score where 0 = 0 skills; 1 = 1 skill; 2 = 2 skills; 3 = three/four skills, 4 = four/five skills and 5 = six + skills.

##### Social Exclusion (Risk Factor)

Respondents’ perceptions of social exclusion or loneliness in relation to family and peers were measured using the 11-item Argentine Adolescent Loneliness Assessment (AALA) [48] with one alteration—the word ‘colleagues’ was replaced with the word ‘students’ to reflect the demographic being tested (e.g., “*My fellow students leave me out*”). The AALA uses a 6-point scale (1 = *Completely untrue*; 6 = *Completely true*), with higher scores denoting greater social exclusion. The AALA has satisfactory to good internal consistency with coefficients ranging from 0.70 to 0.87 [48].

##### Impulsivity (Risk Factor)

The 15-item Barratt Impulsiveness Scale 15 (BIS-15 (ref. [49] has three 5-item subscales: non-planning (e.g., “*I plan for the future*”), motor (e.g., “*I do things without thinking*”) and attentional impulsivity (e.g., “*I don’t pay attention*”) endorsed on a scale where 1 = *rarely/never* and 4 = *almost always*. Higher total and subscale scores denote greater impulsivity. The BIS-15 has good scale and sub-scale reliability and demonstrated validity [50].

### 2.3. Procedure

The participating school sent an email to all parents of students in Years 7 to 10 with details about the study, an assurance of their child’s anonymity, and details of how to opt out of the study. Follow-up emails and the school newsletter then provided the same information. Prior to data collection, a list of students whose parents had opted out of the study (13 in total) was collated, to ensure the relevant students did not participate in the study.

On the days of testing, teachers were provided with an instruction sheet about how to administer the survey and a list of students that were not permitted to participate. Wellbeing staff and the researcher were also in attendance or close by. Students were invited to complete the study in class, with non-participants provided an alternative task deemed by teachers as not unpleasant, to avoid perceived coercion. Participating students then gave their written informed consent, were asked to sit apart from each other, and were provided with a link to the online survey, which was completed on their laptop computers. The demographic questions and IGDT-10 questions were presented in sequential order, but the remaining scales were presented in a randomised order to prevent fatigue effects.

### 2.4. Data Analysis

A-priori power analyses had indicated that the current sample was sufficient for all analyses, with 800 participants needed to detect an effect of *r* = 0.01 at *p* < 0.05 with 80% power. The analytic strategy was to look first at univariate predictors of both the IGD symptom severity score and whether or not participants had reached threshold for IGD diagnosis. Regression analyses using backwards elimination were then used to ascertain the model comprising the strongest predictors for both outcomes, entering only variables that were significant in the bivariate analyses. Finally, risk and protective factors were tallied for each participant, and trend analyses were used to ascertain whether risk for IGD and IGD symptom severity grew as risk factors accumulated, or reduced as protective factors accumulated.

## 3. Results

All scales had adequate to excellent internal consistency, barring parental boundaries, which had lower reliability, possibly due to it consisting of just three items (see Table 1 for reliability scores and descriptives). The two key dependent variables were the numeric score for the IGD-10 (IGD symptom severity) and the categorical variable of meeting IGD diagnostic criteria or not. 24 participants met IGD criteria, giving an IGD prevalence rate of 2.8% in this sample. It is noteworthy that the mean score for IGD severity was 4.17 out of 20, suggesting that most people in this sample had low levels of IGD symptomology. It is also noteworthy that those who met criteria for IGD had significantly higher IGD symptom severity, social exclusion and impulsiveness, and significantly lower self-esteem, control of their environment, parental attachment, family connectedness, and self-control.

### 3.1. Risk and Protective Factors

To test hypothesis 1, simple regression analyses were conducted to indicate the bivariate relationships between IGD symptom severity, meeting IGD criteria, and the various predictors (note that a table of correlations is provided in Appendix B). All participants were included in analyses of symptom severity, as all participants could be scored on that continuum. The sub-group of participants who met IGD criteria were then analysed separately. Simple linear regression was used for the numeric variable IGD symptom severity, and binary logistic regression for the categorical variable “meets IGD criteria” (see Table 2). Across both outcomes, higher levels of self-esteem, control over one’s environment, parent/family connectedness, secure attachment, and self-control were protective factors; and higher levels of social exclusion and impulsivity were risk factors. Being female, an older teen and having more areas of mastery were also protective in terms of IGD symptom severity scores only. In this sample, males tended to have higher IGD symptom severity scores than females, and more males than females met IGD criteria (15 vs. 9) although the latter difference did not reach statistical significance.

To test hypothesis 2, separate regressions using backward elimination were conducted to ascertain the model with the strongest predictors of IGD symptom severity and meeting IGD criteria (see Table 3). All VIF values were below four for both analyses and therefore multicollinearity was not problematic. The final IGD symptom severity model included four protective factors (being female, an older teen, and having greater self-control and control over the external environment) and one risk factor, greater levels of social exclusion. The model overall had significant fit; *F*(5, 815) = 77.99, *p* < 0.001, R^2^ = 0.33, adjusted R^2^ = 0.32. The final model for meeting IGD criteria had just two strong predictors: higher self-control being protective and greater social exclusion causing risk. The diagnostic accuracy of the final IGD model was moderate, with an area under the curve (AUC) analysis of the receiver operating characteristic curve yielding an AUC score of 0.86.

### 3.2. Cumulative Effects of Risk Factors

To ascertain the cumulative effect of risk and protective factors (hypotheses 3–5), it was first necessary to establish the threshold at which scores denoted risk or protection, whilst being cognisant that variables with scores on a continuum typically indicated risk at one extreme and protection at the other. Established thresholds were used for self-esteem (<15 = risk; >25 = protective) [43] and impulsivity (>36 = risk; <26 = protective) [49], while the strategy of Gentile and Bushman was used for the other continuous variables—risk was assigned to scores in the highest risk quartile and protection assigned to scores in the highest protection quartile [36]. Three scores were then calculated for each participant. For risk, each variable was given a score of either 1 (≥the risk cut-off) or 0 (<the risk cut-off), with a total risk score then tallied. For protection, the same strategy was used. A ‘net-risk’ score was then calculated by subtracting the protection score from the risk score, whereby a positive score denoted net-risk (i.e., more risk factors than protective factors) and a negative score net-protection (more protective factors than risk factors).

**Figure 1:**
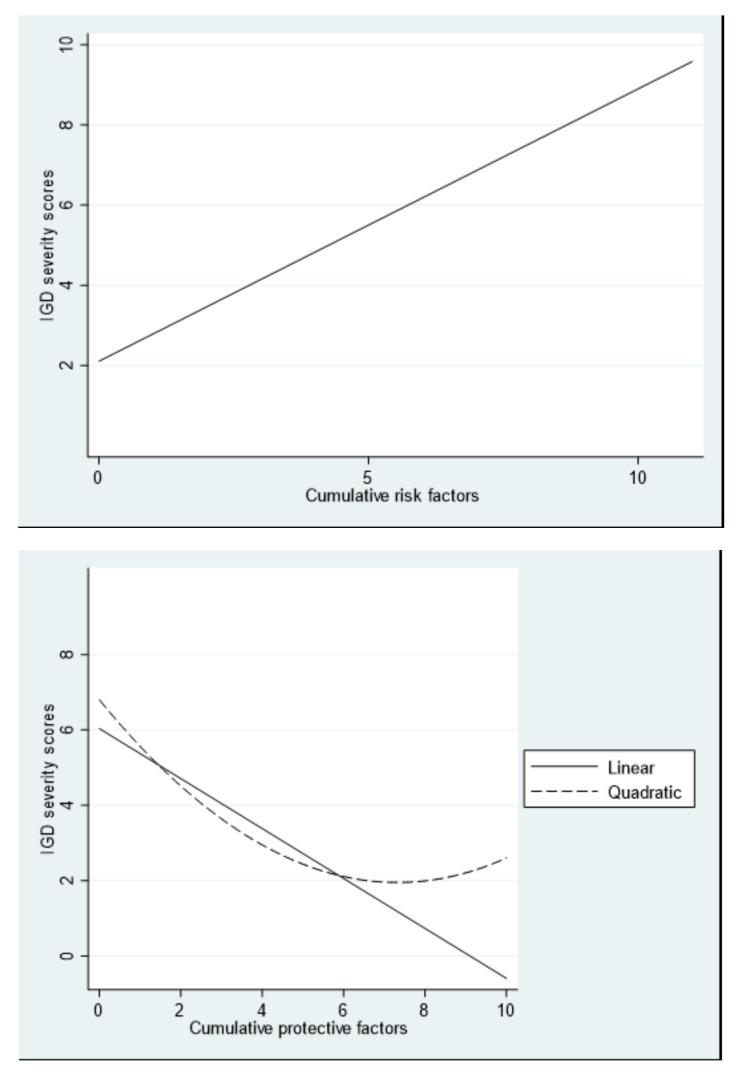

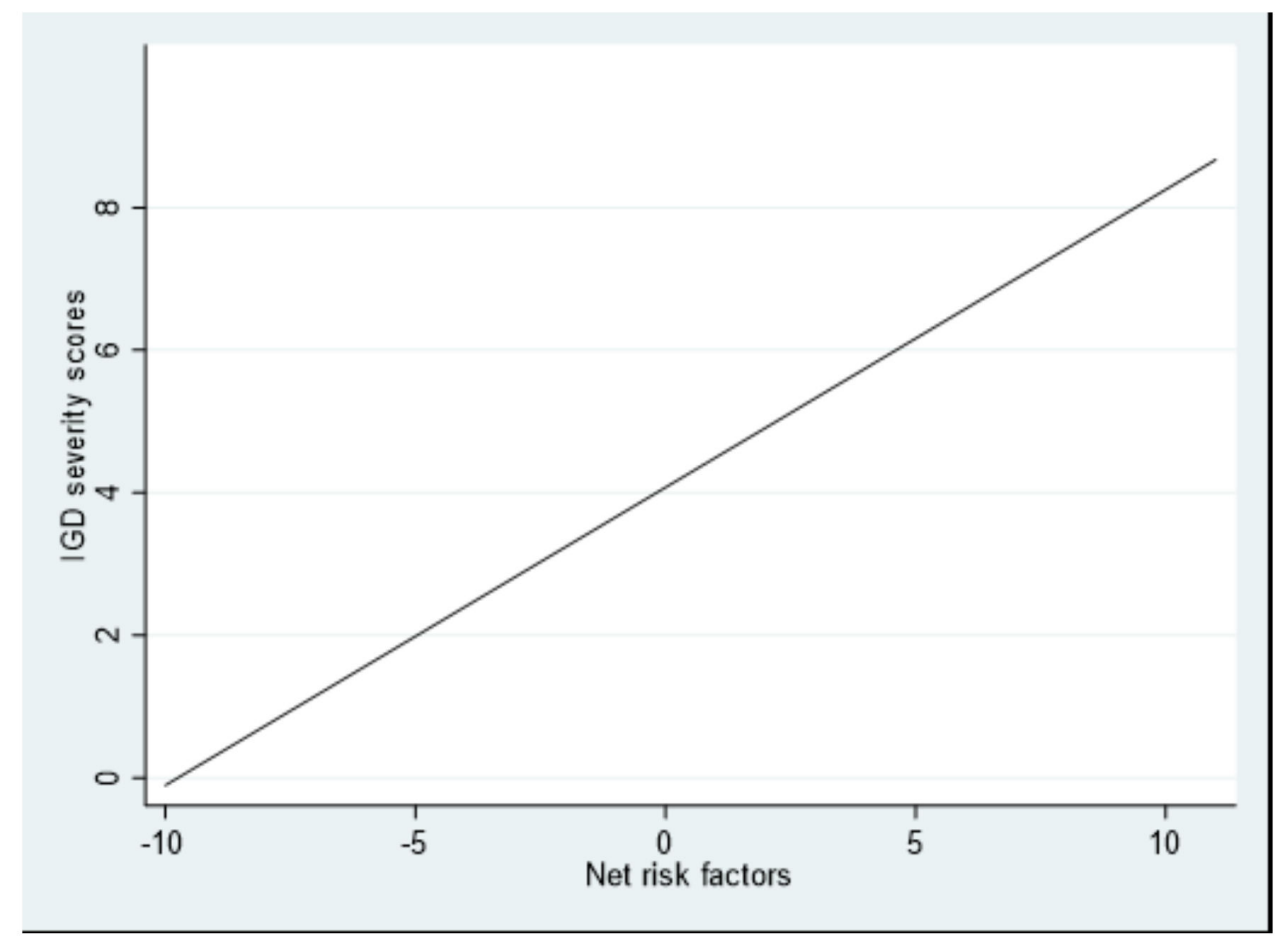
Significant linear/curvilinear relationships between IGD symptom severity scores and risk factors (**top**), protective factors (**middle**), and net-risk factors (**bottom**).

Trend analyses were conducted for the numeric outcome variable, IGD symptom severity (see Figure 1). Linear and quadratic curves were fit for all models (see Table 4 for coefficients for all models). As risk factors accumulated, risk for IGD increased in a linear fashion (*F*(1,864) = 158.39, *p* < 0.001, R^2^ = 0.16). Although the overall model including the quadratic trend was also significant (*F*(2,863) = 79.14, *p* < 0.001, R^2^ = 0.16), the quadratic term itself was non-significant and too small to be meaningfully interpreted. As protective factors accumulated, risk for IGD decreased in a linear fashion (*F*(1,864) = 139.98, *p* < 0.001, R^2^ = 0.14). The overall model including the quadratic term was likewise significant, (*F*(2,863) = 79.41, *p* < 0.001, R^2^ = 0.16), as was the quadratic term itself, suggesting that the protective effect may plateau at approximately six factors. In terms of net-risk factors, there was a significant linear trend (*F*(1,864) = 193.86, *p* < 0.001, R^2^ = 0.18). Although the overall model including the quadratic trend was significant (*F*(2,863) = 98.75, *p* < 0.001, R^2^ = 0.19), the quadratic trend itself was non-significant.

In order to determine whether an accumulation of risk/protective factors was linked to the risk of meeting criteria for IGD, a series of logistic regression analyses was conducted. The odds of meeting criteria for IGD were 1.72 times greater for every added risk factor (B = 0.54, SE = 0.09, OR = 1.72, *p* < 0.001). In contrast, the odds of meeting criteria for IGD markedly decreased for every added protective factor (B = −0.59, SE = 0.17, OR = 0.56, *p* = 0.001). When the net-risk score was used, the net-risk factor score significantly predicted the incidence of IGD (B = 0.37, SE = 0.07, OR = 1.45, *p* < 0.001), with the odds of meeting criteria for IGD, compared to not meeting criteria, being 1.45 times greater for every added net-risk factor.

Further, an independent samples *t*-test revealed that those who met criteria for IGD (*M* = 4.54, SD = 3.19) had a net-risk score over 40 times greater than those that did not meet criteria, *M* = 4.54 (SD = 3.19) vs. *M* = 0.11 (SD = 3.67); *t*(864) = −5.86, *p* < 0.001, Cohen’s d = 1.29.

Together, analyses suggest that an accumulation of risk factors has a profound impact on whether or not a person from this sample met criteria for IGD.

## 4. Discussion

The 2.8% IGD prevalence rate in this sample was similar to the 3.1% prevalence rate found by King and Delfabbro in a 2017 Australian study using a similar sample [7], and is consistent with suggestions that around 1–3% of adolescents meet IGD criteria in Western countries [9]. Males were significantly more likely to have higher IGD symptom severity scores, and more males met clinical criteria for IGD, although in this smaller sub-group this difference did not reach significance. These findings are consistent with numerous studies that have found a greater male preponderance in those with problematic levels of video game use [15,18]. Age effects in the literature are inconsistent, and it is difficult to interpret the age data here, where effects were small or non-significant. Younger participants were somewhat more likely to have higher IGD symptom severity scores, but there was a more even spread in terms of age for those who met clinical criteria for IGD.

Hypothesis one, that the factors measured in the study, at the univariate level, would (a) predict IGD symptom severity scores (and presumably whether or not there is a greater likelihood of PVGU) and (b) predict meeting IGD diagnostic criteria, was mostly supported. All but two of the factors tested in this study significantly impacted the severity of key IGD symptoms at the bivariate level, and a majority of factors significantly predicted whether or not participants met IGD criteria. Significant factors came from all three theorised classes of risk: dysexecutive function, needs unmet offline, and an unhelpful family environment.

As predicted in hypothesis two, the strongest predictors in multiple regression analyses included factors from both unmet needs and dysexecutive function. The strongest predictors that spanned both outcomes were feeling socially excluded and having low self-control, with impulsivity also being a robust predictor across both groups, and having greater perceived control over one’s external environment being a significant predictor for IGD symptom severity scores. These findings are consistent with several past studies. For example, executive dysfunction and self-regulation deficits have been demonstrated in various addictive behaviours [51] as well as in screen-based addictions [23]. Indeed, when Billieux et al. examined the clustered characteristics of three types of disordered gamer identified in their study, all three had issues around elevated impulsivity and behaviour control [22]. In addition, the need to belong is powerful [52], and it is common for those with PVGU or IGD to report loneliness, deficits in offline social relationships and social anxiety [16,24,28,29]. Video gamers with fewer or poorer offline relationships may be repeatedly drawn to seek peer acceptance in the more predictable/controlled online gaming social environment, where a narrower range of social skills may be required and easy withdrawal is possible when socially anxious.

In terms of other needs-based variables that were also significant predictors of both IGD symptom severity and meeting IGD criteria, having little control over one’s offline environment may motivate an individual to keep playing video games because they bolster a sense of personal control and power [9]. Similarly, playing video games is likely to appeal to those low in self-esteem because effort usually translates into reward/success.

With regards to family factors, low family warmth/connectedness and a poorer quality parent-child attachment were significant univariate predictors of both outcomes, although neither were significant in the final models. Two other studies have reported similar findings [7,35], and it may be that poor family function may lose significance in multivariate models of PVGU and IGD predictors because a poor family environment may result in unmet needs and self-control deficits, thus causing substantial shared variance in models that also include unmet needs and self-control. It may be valuable for future longitudinal studies to address whether there is a mediational relationship whereby a dysfunctional family environment fosters unmet needs and self-control problems, with these in turn predisposing the person to PVGU and IGD.

A key finding of this study was that, in line with hypothesis three, an accumulation of risk factors increased IGD symptom severity scores (and thus the likelihood of PVGU and IGD) in a linear fashion. Similarly, they increased the odds of meeting IGD criteria. As hypothesised (H4), the opposite was also true, with protective factors systematically reducing IGD symptom severity scores as they accumulated, and reducing the odds of meeting IGD criteria.

It is important to note that the actual IGD risk for individuals is reflected in the interplay of both risk and protective factors, as every individual who plays games has both. Thus, the impact of net-risk is particularly salient when applying these findings to the ‘real world’. In this light, it seems important that, in line with Hypothesis 5, net-risk trends also showed the same pattern. A profile of elevated net-risk substantially increased the risk of having higher levels of IGD symptomology in a linear fashion. Similarly, the odds for meeting IGD criteria increased with every additional net risk factor. Indeed, in this sample, those who met IGD criteria had a mean net-risk score over forty times that of those who did not.

Taken together, these findings suggest that risk and protective factors, and net-risk, play a key role in degree of IGD symptomology and the development of IGD in middle teenagers. Thus, a risk and resilience approach may offer valuable insights into both the developmental causes and treatment of PVGU and IGD [13].

In terms of the latter, these findings suggest that adolescents with executive dysfunction, such as poor self-regulation and impulsivity, and/or whose needs are not met in the offline world, and/or who have a disconnected family environment lacking in warmth, may have an elevated risk of developing IGD or more severe IGD symptomology, especially if there are fewer factors providing protection. In addition, the risk for IGD or more severe IGD symptomology would increase as net-risk factors accumulate. Thus, by identifying risk factors across these domains early, it may be possible to also identify children at risk of Problematic Video Game Use or IGD early, and to establish interventions that prevent their onset, an approach in line with better mental health practice [53].

Clinically, it is also important to note that every individual who plays video games will have a unique constellation of factors that increase and decrease risk related to video game use, with the interplay of these factors (with each other and with other factors), and the resulting net-risk, determining the likelihood of Problematic Video Game Use or IGD [37]. If clinicians can identify and remediate risk factors, the likelihood of developing/maintaining Problematic Video Game Use or IGD seems much smaller. For example, in this study those with IGD had an average net-risk score of around five. If just a few risk factors could be remediated (e.g., offline friendships established, self-esteem bolstered through offline achievements, self-regulatory ability improved), then the net-risk could be substantially reduced. Thus, clinical interventions that focus on identifying and reversing those risk factors that can realistically be changed may turn out be have substantial therapeutic benefit.

Finally, parental boundaries on video game use did not predict reduced IGD symptom severity or reduced likelihood of meeting IGD criteria. If this finding can be replicated, it may suggest that putting limits on time spent video gaming is unlikely to work as a standalone prevention approach, and should be supplemented by strategies related to other factors underpinning Problematic Video Game Use and IGD.

### Limitations and Future Research

The key limitation to this study is that even with nearly a thousand initial participants, the small number of clinical-level IGD participants (2.8%) makes confident interpretation of the results for that subgroup difficult. In addition, the cross-sectional nature of the study precludes making conclusions related to the causality of the predictors. Clearly, more longitudinal studies are needed to understand the development of IGD, and the causal role that the various risk and protective factors play. We also acknowledge the superiority of multiple diagnostic measures over and above simple self-report, including parent assessments, performance-based measures and clinical observations. Whilst the collection of such data was not possible in the current large sample of school children, smaller studies examining the same issues should consider using multiple diagnostic techniques. In terms of measures, the results suggest that social potency, the ability to be socially dominant and persuasive, may not be important in terms of IGD. Future studies looking at risk and protective factors may be better served by looking at basic social competence, a related skill that has previously been found as lacking in both PVGU and IGD [19,28]. Finally, further research examining IGD from a risk and resilience approach would also benefit from testing a larger and more diverse sample.

## 5. Conclusions

This study provides evidence that unmet needs in off-screen life (e.g., social isolation, low control over one’s environment), in tandem with poor executive function (e.g., low self-control), together are potent predictors for PVGU and IGD. Importantly, as such risk factors accumulate in the individual without being offset by protective factors, the risk of PVGU and IGD may grow concomitantly. Taken together, the results of this study suggest that a risk and resilience approach to IGD may be valuable both for understanding the development of IGD and for informing clinical interventions.

## Figures and Tables

**Table 1:** Descriptives for overall sample and those who met IGD criteria; Scale reliability.

Variable	Overall Sample		Meet IGD Criteria				
	Mean	SD	Mean	SD	*t*	*p*	α
IGD symptom severity	4.17	3.64	14.08	2.12	−15.24	<0.001	0.81
Self-Esteem	28.12	6.33	22.13	7.42	4.77	<0.001	0.90
Social Exclusion	24.68	12.89	36.58	16.57	−4.64	<0.001	0.94
Impulsiveness	32.23	7.29	39.04	6.47	−4.70	<0.001	0.82
Environmental Control	32.13	6.64	25.21	8.52	5.26	<0.001	0.80
Mastery Demonstrated	3.08	1.26	2.59	1.22	1.86	0.063	-
Parental Attachment	36.75	7.20	30.83	8.20	4.12	<0.001	0.87
Parental Boundaries	1.67	1.09	1.52	1.16	0.68	0.499	0.57
Parent-Family Connectedness	4.30	0.78	3.69	1.06	3.89	<0.001	0.92
Self-Control	42.35	9.09	31.08	6.85	6.30	<0.001	0.81
Social Potency	6.54	3.07	7.13	3.78	−0.95	0.345	0.69

**Table 2:** Univariate regression analyses for IGD symptom severity and IGD.

Variable	IGD Symptom Severity			Meets IGD Criteria		
	**B**	**SE**	**β**	**B**	**SE**	**OR**
Gender	−2.75 ***	0.23	−0.38	−0.25	0.43	0.78
Age	−0.23 *	0.10	−0.08	0.21	0.17	1.23
Self-Esteem	−0.10 ***	0.02	−0.17	−0.15 ***	0.03	0.86
Social Exclusion	0.06 ***	0.01	0.023	0.06 ***	0.01	1.06
Impulsivity	0.10 ***	0.02	0.21	0.11 ***	0.03	1.12
Control over environment	−0.13 ***	0.02	−0.24	−0.15 ***	0.03	0.86
Mastery ^a^	−0.30 **	0.10	−0.11	−0.30	0.16	0.74
Attachment	−0.10 ***	0.02	−0.20	−0.10 ***	0.03	0.90
Boundaries ^b^	−0.05	0.12	−0.01	−0.13	0.19	0.88
Family Connectedness	−0.73 ***	0.16	−0.16	−0.70 ***	0.19	0.50
Self-control	−0.14 ***	0.01	−0.36	−0.15 ***	0.03	0.87
Social potency	−0.05	0.04	−0.04	0.06	0.07	1.07

Note. N = 866. IGD criteria (0 = did not meet criteria for IGD, 1 = met criteria for IGD); Gender (0 = Male, 1 = Female); OR = odds ratio; ^a^ N = 816, ^b^ N = 674; * *p* < 0.05, ** *p* < 0.01, *** *p* < 0.001.

**Table 3:** Regression models predicting IGD symptom severity and meeting IGD criteria using backward elimination.

Variable	B	SE	95% CI	β	*t*	*p*	% var.
	**IGD symptom severity**						
Gender	−3.10	0.21	[−3.51, −2.68]	−0.43	−14.52	<0.001	17.56
Age	−0.32	0.09	[−0.49, −0.16]	−0.11	−3.75	<0.001	1.16
Control over environ.	−0.04	0.02	[−0.08, −0.004]	−0.08	−2.18	0.030	0.40
Self-control	−0.12	0.01	[−0.15, −0.09]	−0.30	−8.91	<0.001	6.60
Social exclusion	0.04	0.01	[0.02, 0.05]	0.12	3.58	<0.001	1.06
	**Meets IGD Criteria**						
	**B**	**SE**	**95% CI OR**	**OR**	**Wald**	***p***	
Self-control	−0.13	0.03	(0.83, 0.92)	0.88	24.34	<0.001	
Social exclusion	0.04	0.02	(1.01, 1.07)	1.04	6.58	0.010	

Note. % var. = unique variance explained; OR = Odds ratio.

**Table 4:** Linear and Quadratic coefficients for curve estimation.

Model	Term	B	SE	β	*t*	*p*
**Cumulative risk factors**						
Linear only	Linear	0.68	0.05	0.39	12.59	<0.001
Linear and	Linear	0.72	0.17	0.42	4.27	<0.001
quadratic	Quadratic	−0.005	0.02	−0.02	−0.23	0.820
**Cumulative protective factors**						
Linear only	Linear	−0.66	0.06	−0.37	−11.83	<0.001
Linear and	Linear	−1.33	0.17	−0.75	−7.66	<0.001
quadratic	Quadratic	0.09	0.02	0.39	4.04	<0.001
**Model**	**Term**	**B**	**SE**	**β**	***t***	***p***
**Net-risk factors**						
Linear only	Linear	0.42	0.03	0.43	13.92	<0.001
Linear and	Linear	0.41	0.03	0.42	13.70	<0.001
quadratic	Quadratic	0.01	0.01	0.06	1.77	0.080

## Data Availability

The data presented in this study are available on request from the corresponding author. The data are not publicly available due to restrictions on who can view the data applied by the human research ethics committee that approved this research.

## Appendix A

**Feeling in Control of One’s Own Environment Scale**

*Response scale:* 1 = Strongly disagree; 2 = Slightly disagree; 3 = Disagree; 4 = Agree; 5 = Slightly agree; 6 = Strongly agree.

*Instructions*. Read each statement carefully, but don’t spend too much time deciding on the answer. Indicate to what extent you agree with the following statements.

1. I feel in control of most areas of my life.
2. I find it hard to manage the demands of school (R).
3. I have the power to manage the demands of school.
4. I often feel helpless when dealing with the problems in my life (R).
5. I am able to influence the people around me.
6. I have little power over the things that happen to me (R).
7. If I want to do something, most of the time I can make it happen.
8. Other people determine most of what I can and cannot do (R).
9. My family includes me in decision making.
10. Overall, I feel in control of the world around me.

## Appendix B

**Table A1:** Correlation Matrix.

No	Variable	1	2	3	4	5	6	7	8	9	10	11	12	13
1	IGD symptom severity													
2	IGD Criteria	0.46												
3	Weekly Gaming Time	0.60	0.29											
4	Mastery	−0.11	−0.07	−0.10										
5	Self-esteem	−0.17	−0.16	−0.04	0.25									
6	Impulsiveness	0.21	0.16	0.13	−0.21	−0.37								
7	Self-control	−0.40	−0.21	−0.21	0.23	0.45	−0.69							
8	Parental attachment	−0.20	−0.14	−0.07	0.18	0.55	−0.42	0.50						
9	Social potency	−0.04	0.03	−0.08	0.18	0.18	0.01	0.04	0.02					
10	Control environment	−0.24	−0.18	−0.12	0.26	0.68	−0.43	0.49	0.54	0.24				
11	Social Exclusion	0.23	0.16	0.09	−0.13	−0.59	0.24	−0.34	−0.43	−0.11	−0.53			
12	Family Connectedness	−0.16	−0.13	−0.07	0.18	0.48	−0.33	−0.39	0.77	0.03	0.47	−0.40		
13	Family Boundaries	−0.01	−0.03	−0.15	0.14	0.18	0.17	0.24	0.18	0.05	0.12	−0.14	−0.15	
14	Age	−0.08	0.04	−0.01	−0.11	−0.06	0.03	−0.09	−0.09	0.04	0.03	−0.03	−0.06	−0.32

Note. Significant correlations in bold. All correlations where *r* > 0.12 are significant at *p* < 0.001; all correlations between *r* = 0.08 and *r* = 0.12 are significant at *p* < 0.05.
